# Infant Young Child Feeding Practices in an Indian Maternal–Child Birth Cohort in Belagavi, Karnataka

**DOI:** 10.3390/ijerph19095088

**Published:** 2022-04-22

**Authors:** Shweta Khandelwal, Dimple Kondal, Anindita Ray Chakravarti, Soumam Dutta, Bipsa Banerjee, Monica Chaudhry, Kamal Patil, Mallaiah Kenchaveeraiah Swamy, Usha Ramakrishnan, Dorairaj Prabhakaran, Nikhil Tandon, Aryeh D. Stein

**Affiliations:** 1 Public Health Foundation of India, Delhi NCR 122002, India; dimple@ccdcindia.org (D.K.); monica.chaudhry@phfi.org (M.C.); dprabhakaran@phfi.org (D.P.); 2 Centre for Chronic Disease Control, New Delhi 110016, India; 3 Department of Food & Nutrition, Maharani Kasiswari College, University of Calcutta, Kolkata 700073, India; anindita.ray.chakravarti@gmail.com; 4 Department of Home Science, University of Calcutta, Kolkata 700027, India; soumam_dutta@yahoo.com (S.D.); nutribipsa@gmail.com (B.B.); 5 KAHER’s JN Medical College, Belagavi 590010, India; kamalpatil1967@yahoo.co.in (K.P.); mkswamy53@yahoo.co.in (M.K.S.); 6 Rollins School of Public Health, Emory University, Atlanta, GA 30322, USA; uramakr@emory.edu (U.R.); aryeh.stein@emory.edu (A.D.S.); 7 Department of Endocrinology and Metabolism, All India Institute of Medical Sciences, New Delhi 110029, India; nikhil_tandon@hotmail.com

**Keywords:** infant young child feeding practices, diet diversity, anthropometry, complementary foods, South India, breastfeeding

## Abstract

Poor infant young child feeding (IYCF) practices result in malnutrition, poor psychosocial development, poor school performance and less productivity in later life, thereby perpetuating a vicious cycle. The current study aims to characterize the IYCF practices during the first year of life in a maternal–child birth cohort (DHANI) in Belagavi, Karnataka, India. We collected data from the dyad at birth, 6 and 12 months postpartum. We examined dietary diversity among these infants at 12 months using WHO criteria. A total of 902 live births were recorded, and 878 mother–child pairs completed the 12-month follow up. The overall prevalence of early (within 1 h of delivery) initiation of breastfeeding (EIBF) was 77.9%, and that of exclusive breastfeeding (EBF) at 6 months was 52.4%. At 12 months, most (90%) infants were breastfed, while 39% also received formula. The large majority (94.4%) of infants met minimum meal frequency (MMF), but only 55% of infants were receiving a minimum acceptable diet (MAD). The mean dietary diversity (DD) score was 4.7 ± 1.1. Only 21.9% of infants consumed egg and/or flesh food. A large proportion (33.8%) of infants received no vegetables and/or fruits till 12 months of age. Consumption of sweet beverage was 4.8%, but consumption of ultra-processed foods high in trans-fats, sugars and salt was high (85.8%). High-quality, sustainable and scalable interventions to enhance knowledge and support positive behaviour change for adopting and implementing better IYCF practices may be urgently needed in low- and middle-income group settings to improve diet diversity and overall nutritional intake amongst young children.

## 1. Introduction

Undernutrition is responsible for about 45% of deaths among children under 5 y worldwide, being an important etiological factor that increases vulnerability to diarrhea, malaria and pneumonia [1]. Poor infant and young child feeding (IYCF) practices, especially during the first thousand days (birth to 2 years old), result in malnutrition, poor psychosocial development, poor school performance and less productivity in later life, thereby setting up a vicious cycle [2]. An analysis showed that appropriate breastfeeding and complementary feeding practices could alone prevent under-five deaths by 19% [3]. Although improvements have been made, dietary quality remains suboptimal for infants and young children globally and has not improved much in the last decade [4]. In urban and rural regions, respectively, only about 39% and 23% of children under 5 years of age receive a minimally diversified diet [4].

The World Health Organization/United Nations Children’s Emergency Fund (WHO/UNICEF) has operationalized IYCF guidelines [5,6]; the core recommendations include early (within one hour of birth) initiation of breastfeeding (EIBF), exclusive breastfeeding for the first six months (EBF), continued breastfeeding for up to two years (CBF) and the introduction of nutritious and safe complementary food at six months of age. It is extremely important to ensure a Minimum Acceptable Diet (MAD), which is defined as a diet having Minimum Dietary Diversity (MDD) and Minimum Meal Frequency (MMF) [5]. MDD is an indicator of micronutrient density, whereas MMF reflects the adequacy of energy intake. Recently, the WHO and UNICEF have introduced additional indicators, including whether the child was ever breastfed, was exclusively breastfed for the first two days, received mixed milk feeding under six months, consumed egg and/or flesh food, consumed no vegetable or fruits, consumed sweet beverage or consumed foods considered unhealthy [5,6]. The WHO/UNICEF’s Global breastfeeding scorecard is also another advocacy tool to call for improving, monitoring and tracking robust action in the breastfeeding efforts by each country. This again highlights the significance of investing in better IYCF practices to positively shape children’s growth and development.

In India, the burden of both childhood undernutrition and overnutrition are increasing along with multiple micronutrient deficiencies, also known as “hidden hunger” [4,7,8]. The National Family Health Survey-4 (NFHS-4; 2015-16) data show that the all-India prevalence of EIBF was 41.6% and EBF was 54.9%. Less than half (42.7%) of children aged 6–8 months were receiving solid or semi-solid food along with breastfeeding, and only 9.6% of children aged 6–23 months were receiving an adequate diet [9]. The Comprehensive National Nutrition Survey (CNNS) of 2016–2018 [10] has reported similar data. The prevalence of EIBF was 57% of children under 2 years, 58% of infants were exclusively breastfed and 83% of children received continued breastfeeding at one year old. The timely introduction of complementary food was 53%. MDD, MMF and MAD were achieved by only 42%, 21% and 6% of children aged 6–23 months. However, since most of the studies conducted have been cross-sectional in nature and longitudinal data are scanty, high-quality studies highlighting the regional variation in IYCF and understanding specific reasons or attributes to inform and strengthen practices are urgently warranted.

In the current study, we characterize the breastfeeding patterns and IYCF practices at 6 and 12 months in a longitudinal follow-up of a maternal–child birth cohort in Belagavi, Karnataka, India [11]. We also examine the dietary diversity among these infants at 12 months. The association among maternal sociodemographic factors, health conditions and breastfeeding and child’s dietary diversity have also been examined in the paper. The findings may inform local governance, plan required interventions and help strengthen gaps where applicable.

## 2. Methods

Women were participants in a double-blind, parallel-group, randomized, placebo-controlled trial consisting of 957 pregnant women aged 18–35 years from ≤20 weeks gestation through 6 months postpartum with 400 mg/d algal-derived Docosahexaenoic Acid (DHA) or placebo, referred to as the DHANI trial for short [11]. The first mother was enrolled on the 6th Jan 2016 and the last one on the 31st Aug 2017. The mother–infant dyads were followed from recruitment (less than equal to 20 weeks gestation) through 12 months of age, and the data collection ended in April 2019. Ethical clearances for the DHANI study were obtained from the Institutional Ethics Committee, Jawaharlal Nehru Medical College (JNMC), Public Health Foundation of India (No: TRC-IEC-261/15.1), and the Centre for Chronic Disease Control (CCDC). The trial is registered on both ClinicalTrials.gov (Identifier NCT03072277) and Ctri.nic.in (Identifier CTRI/2017/08/009296).

### 2.1. Data Collection

Data on the pregnant women’s socio-demographic and health profile (age, education, occupation, household income, family size and structure, parity, obstetric history, etc.), anthropometric measurements (height, weight, mid-upper arm circumference) and dietary intake were recorded at baseline. The delivery details such as type of delivery, child anthropometry, hospitalization, etc., were also collected. The method collection details have already been published elsewhere [11]. In short, trained personnel interviewed pregnant women (at ≤20 weeks of gestation) and noted their socio-demographic details and anthropometry. Information on breastfeeding and complementary feeding was collected from the mother at 1, 6 and 12 months postpartum by trained research personnel. Specific information on colostrum, pre-lacteal feeding, reasons for not breastfeeding (as applicable), continued breastfeeding, etc., were gathered using questionnaires. At 12 months, a Food Frequency Questionnaire (FFQ) indicative of the food groups consumed by the infant over the past 6 months was completed. Food groups covered in the Food Frequency Questionnaire are provided in Supplement Materials Box S1. A 24 h dietary recall for the infant was also administered at 12 months. Both these tools were validated during piloting [11]. Data were coded and assigned to the following categories: breast milk, grains, roots, tubers and plantains, pulses (beans, peas, lentils), nuts and seeds, dairy products (milk, infant formula, yogurt, cheese), flesh foods (meat, fish, poultry, organ meat), eggs, vitamin-A-rich fruits and vegetables and other fruits and vegetables.

### 2.2. Derived Variables

We computed the set of WHO IYCF indicators, including EIBF: early initiation of breastfeeding within 1 h of birth; EBF: exclusive breastfeeding for the first 6 months; CF: initiation of complementary feeding at 6 months; CBF: continued breastfeeding at 12 months, attainment of MDD, MMF and MAD, attainment of MMFF: minimum milk feeding frequency for non-breastfed children, EFF: egg and/or flesh food consumption, SwB: sweet beverage consumption, UFC: unhealthy food consumption, ZVF: zero vegetables or fruit consumption and BoF: bottle feeding at 12 months [6]. The definitions of each are provided in Supplement Materials Box S2. The 24 h dietary recall data were used for calculating the dietary diversity scores. If a food group was consumed, a score of ‘1’ was assigned, and if not, a score of ‘0’ was assigned. The total scores were summed up to obtain the diet diversity (DD) scores for each infant.

### 2.3. Statistical Analysis

Continuous variables were reported as mean (standard deviation), and categorical variables were reported as number (percent). Bivariable association of EBF with mother’s demographic and child characteristics was performed using the chi-square test for categorical variables and t-test for continuous variables. The log binomial regression was used to determine the strength of association of EBF with mothers’ age, education, parity, type of delivery and hospitalisation of a child reported at 1 month, 6 months or 12 months. The unadjusted and adjusted relative risk with a 95% confidence interval (CI) was reported. The model was adjusted for the mother’s age, education, income, parity, type of hospital delivery and hospitalisation of the child reported at 1 month, 6 months or 12 months.

The difference in mean child weight, height and head circumference at 6 and 12 months by EBF was assessed using a t-test. The mean difference with a 95% confidence interval was reported. Multiple linear regression was used to determine the association of child anthropometrics, i.e., birth weight, height and waist circumference, as the outcome variable and exclusive breastfeeding as the exposure variable. We reported the β coefficient adjusting for each of the factors, i.e., mother’s age, mother’s education, household income, mother’s BMI, parity, delivery type and treatment group (the intervention group had received 400 mg DHA/d from enrolment, i.e., less than 20 weeks gestation to 6 months postpartum).

We assessed the association of the DD score with the mother’s age, education, income, parity, type of delivery and breastfeeding exlusivity. The mean difference between DD score with mothers’ age, education, income, parity and type of delivery was assessed using a one-way analysis of variance and *t*-test.

All statistical analysis was carried out using Stata 16.0 (MP) version.

## 3. Results

There were 902 live births of 957 randomized women. Baseline characteristics of mothers are presented in Table 1. The CONSORT diagram is provided as Supplementary Materials Figure S1. The mean age of the mothers was 23.5 (3.6) years, and the gestational age at enrolment was 15.0 weeks. Most had completed high school (78.9%) and were homemakers (76.1%). There were 902 singleton births, of whom 477 (53%) were males. The infant’s median birth weight, length and head circumference of the infant were 2740.0 g, 47.2 cm and 34.0 cm, respectively. The median APGAR score at 1 min and 5 min was 7.0 and 8.0, respectively.

### 3.1. Early Breastfeeding Practices (at Birth)

Among the 902 women who delivered a live child, 703 (77.9%) reported initiating breastfeeding within 1 h. The data are provided in Table 2. The most frequently reported reasons for the delay in the initiation of breastfeeding were that breastmilk could not be expressed within the first hour (4.2%), and there was a delay in shifting the mother to the maternity ward after delivery (7.7%).

The colostrum was fed to 859 (95.2%) infants. A total of 77 (8.5%) children were reported to receive pre-lacteals. Honey was provided to 3.9% of infants, and sugar water was provided to 3.4% of the infants as pre-lacteal feed.

Prior to discharge, 843 (93.5%) children were exclusively breastfed, with 1% of the mothers providing mixed feeding and another 1.4% providing exclusive formula feeding/expressed milk.

### 3.2. Feeding in the First Month and Complementary Feeding Practices at Six Months

**Figure 1:**
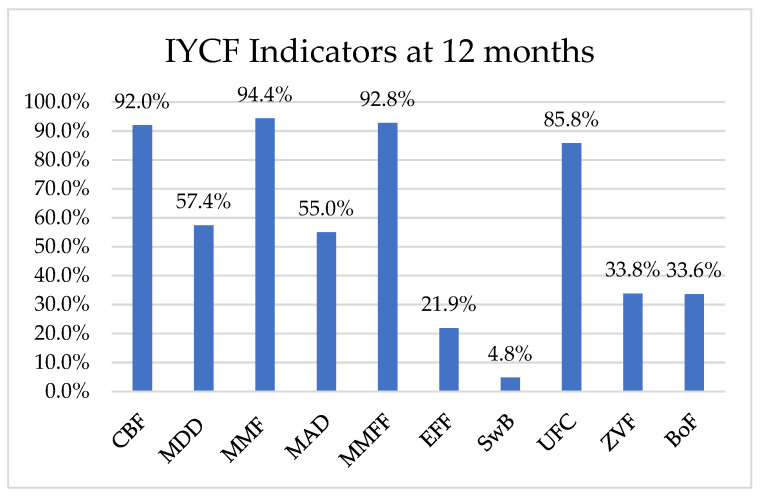
IYCF Indicators at 12 months. CBF: Continued breast feeding; MDD: Minimum Dietary Diversity; MMF: Minimum Meal Frequency; MAD: Minimum Acceptable Diet; MMFF: Minimum Milk Feeding Frequency for Non-Breastfed Children; EFF: Egg and/or Flesh Food Consumption; SwB: Sweet Beverage Consumption; UFC: Unhealthy Food Consumption; ZVF: Zero Vegetable or Fruit Consumption; BoF: Bottle Feeding.

About 774 (85.8%) children were reported to be exclusively breastfed at one month, and 427 (47.3%) continued this exclusive breastfeeding till six months. In total, 321 (35.6%) infants were introduced complementary foods during their sixth month. About 9.3% (*n* = 84) of mothers reported giving one complementary feed per day; 18% (*n* = 160) said they provided it twice; and the rest provided three or more times per day. The median age for introducing complementary feed was 5.5 months, and the frequency of complementary feed was reported as either once, twice a day or more than three times at 9.3%, 17.7% and 8.6%, respectively. The reported consistency of the complementary foods ranged between thin and medium. About 88.8% of the mothers reported practicing responsive feeding at 6 months. Finally, 71 mothers reported providing bottle-feeding at 6 months. IYCF indicators at 12 months are provided in Figure 1.

### 3.3. Feeding Practices at Twelve Months

Breastfeeding was being continued for 90% of infants at 12 months. The proportion of infants meeting MMF and MDD was 94.4% and 57.4%, respectively. Further, only 55% infants were receiving a MAD. The mean DD score was 4.7 ± 1.1. Around 93% of non-breastfed infants achieved MMFF. Only 21.9% of infants consumed egg and/or flesh food. A large proportion (33.8%) did not consume vegetable and/or fruits. The consumption of sweet beverages was low (4.8%) but very high (85.8%) for ultra-processed foods high in trans fats, sugars and salt. Prevalence of bottle feeding at 12 months was 33.6%.

### 3.4. Association of Exclusive Breastfeeding at 6 Months with Maternal and Infant Characteristics

Mothers with secondary education were more likely to breastfeed at 6 months than those who were less educated (56.4% vs. 47.0%, *p*-value = 0.006). The mothers who had normal delivery were more likely to exclusively breastfed compared to those who had caesarean deliveries (67.4% vs. 58.0%, *p*-value = 0.005). Infants who were exclusively breastfed at 6 months were also less likely to be hospitalized when compared to those who were not exclusively breastfed (7.3% vs. 11.3%, *p*-value = 0.044). Detailed data are provided in Table 3.

### 3.5. Association of Exclusive Breastfeeding at 6 Months with Child Weight, Height and Head Circumference at 6 and 12 Months

There were no significant differences in child anthropometry (weight, height, and head circumference) when comparing exclusively breastfed infants to those who were not exclusively breastfed at 6 or 12 months of age. The detailed data are provided in Table 4.

### 3.6. Association of Diet Diversity (DD) Score with the Mother’s Characteristics

The mean DD score was 4.66 (1.15). DD score was higher among infants in households with monthly income ≥ INR 20,000 as compared to households with incomes in the range of INR 10,000–20,000 and those with household monthly income < INR 10,000 (4.93 (1.17) vs. 4.76 (1.09) vs. 4.55 (1.16), respectively, *p*-value = 0.002). The detailed data are provided in Table 5.

## 4. Discussion

Our analysis of IYCF practices among mother–infant dyads in the DHANI cohort in Belagavi, Karnataka, showed that the overall prevalence for early initiation of breastfeeding (EIBF) within 1 h was 77.9% and that of exclusive breastfeeding (EBF) at 6 months was 52.4%. In another study from Belagavi, 65.9% of mothers reported EBF [12]. A similar study from Wardha, Central India, reported lower percentages of EIBF (41.6%) and EBF (36.6%) [13]. In the capital city of India, these percentages were reported to be even lower (EIBF, 38.8%; EBF, 30.6%) [14]. Only 61% of children under 6 months are exclusively breastfed as per the NFHS-5 (2021) report [15]. In the current study, 33.6% of mothers reported bottle-feeding by 12 months, which is significantly higher than that reported in another study from a similar setting (3.2% bottle-fed) [12]. The global weighted prevalence for 57 LMICs (2010–2018) is around 51.9% for early initiation of breastfeeding, 45.7% for exclusive breastfeeding till 6 months and 83.1% for continued breastfeeding at 1 year [16].

Given that prelacteal meals can cause delays in breastfeeding initiation, as well as hinder the appropriate setup and future success of breastfeeding [17], in our study, only 8.5% of babies were given prelacteal feed, and honey and sugar water were mostly given. This was largely reported due to traditional practices followed by the family. We also found a significant positive association between the mother’s education and EBF till 6 months. A study in Uganda found no association between maternal education and IYCF practices [18]. On the contrary, in Ethiopia, the maternal education status of primary school has been identified as one of the independent predictors of IYCF practices [19]. A review of trends of IYCF in 81 LMICs from 2000–2019 showed a significant increase in early initiation and exclusive breastfeeding across all education categories. This increase has been more pronounced for early breastfeeding and exclusive breastfeeding among women with no formal education and in higher educated women, respectively [20]. In our study too, maternal education positively influenced EBF practice. In addition, household income also emerged as a significant variable that influenced EBF. Other studies also confirm this association.

According to NFHS-5, less than half (45%) of children in Karnataka receive breastmilk and complementary foods at the age of 6–8 months [21]. Infants who were exclusively breastfed in our study till 6 months of age had a mean bodyweight of 6.9 kg and 8 kg at 6 and 12 months, respectively. The infants who were not exclusively breastfed had mean weights of 6.8 kgs and 8.3 kgs at 6 and 12 months, respectively. In the US, infants who were breastfed till 6 months of age had lower fat-free mass, and greater trunk fat mass and body fat percent than in formula-fed infants at 6 months of age [22]. Whether this association holds in Indian children is not known.

In this present study, 33.8% were not fed any vegetables or fruits till about 12 months of age. A majority of infants (85.8%) were also fed at least some ‘unhealthy’ foods. A low proportion of infants met the MDD, defined as consumption of foods from five or more food groups. Another study from Delhi noted that 32.6% of infants aged 6–23 months met their MDD [23]. This was lower than that reported in the present study (57.4% at 12 months). Infants who consume foods 3–4 times per day are considered to meet the MMF indicator. Our study shows 94.4% MMF, which is more than another study from Karnataka reporting 82.8% MMF [24]. NFHS-5 in Karnataka reported 12.8% MAD in 12–17 months, which is significantly lower than our findings of 55% MAD at 12 months of age [15]. Secondary analysis of NFHS-4 data has also found that as low as 10% of children in the 6–23-month age group consume a MAD, and this trend has remained the same for a decade [25]. A study in Ethiopia found that 17% and 72.2% of children aged 6–23 months consumed diets that met the criteria for MDD and MMF, respectively [26]. A study on diet quality of infants aged 6–23 months in 42 LMICs has revealed that wealthier households introduce more diverse foods at earlier ages [27]. Furthermore, a recent study reported that only 21.3%, 56.2% and 10.1% of the 80 LMICs with data on IYCF had prevalence levels above 50% for MDD, MMF and MAD among children aged 6–23 months, respectively [28].

A systematic review on interventions in LMICs has revealed that breastfeeding education interventions can improve the rates of early initiation of breastfeeding by 20%, exclusive breastfeeding (EBF) at 3 months by 102% and EBF at 6 months by 53%. Additionally, when these interventions are led by healthcare professionals, they are more effective [29]. Other interventions that can help promote breastfeeding amongst working women include having crèches at the workplace and flexible working hours and creating public awareness of the dangers of bottle and formula feeding [30].

The results of the present study must be interpreted in light of some limitations. This study was conducted in one community in southern India and may have limited generalizability. Although we used pre-piloted tools and established a good rapport with the enrolled participants (as indicated by high rates of retention and follow-up), the quality of diet data can be influenced by the mother’s recall and social desirability bias. The mother–infant dyads in the DHANI cohort were followed up only till 12 months as per the parent study plan and available funds. The information on the exact measure of ingredients for all the commonly consumed dishes was not available. However, all efforts to capture food groups and frequencies were rigorously carried out by trained staff.

## 5. Conclusions

It can be concluded that in our cohort from Belagavi in South India, compliance with three IYCF recommendations, i.e., the early initiation of breastfeeding within one hour of birth, exclusive breastfeeding for first six months and the introduction of complementary foods at the age of six months is sub-optimal. Our findings show that poor IYCF practices were continued (poor diet diversity, minimum acceptable diet, consumption of high fat, salt and sugary foods, etc.) through the first year of the child’s life. This could be because of poor awareness or lower education of the women regarding IYCF practices. It could also be that the health care workers were not emphasizing or offering sustained support on correct IYCF practices. Most of the women enrolled in the present study were first-time mothers and thus may lack appropriate experience and knowledge of infant feeding. Improvement in IYCF practices necessitates support from multiple stakeholders, including health care workers, family members and friends, peer educators, etc. Community-level campaigns should be organized so as to create mass level awareness amongst the caretakers. Mother and family should be motivated, encouraged, educated and supported regarding proper infant feeding practices so as to sustain and maintain infant health. Appropriate nutritional practices, especially during their first 1000 days (conception to two years), play a pivotal role in determining optimal health and development of children.

## Figures and Tables

**Table 1:** Mother and Child characteristics.

Mother	*n* = 902, *n* (%)
Mother’s age (years), mean (SD)	23.5 (3.6)
Gestation age (week), median (IQR)	15.0 (12.0, 18.0)
Parity	
*Multiparous*	469 (52.0)
*Nulliparous*	433 (48.0)
Education	
*College graduate and above*	163 (18.1)
*High school/Secondary*	712 (78.9)
*Up to primary*	27 (3.0)
Employment Status	
*Working*	216 (23.9)
*Homemakers*	686 (76.1)
Household income per month (INR)	
*<10,000 (USD 133.46)*	216 (23.9)
*10,001 (USD 133.47)–20,000 (USD 266.91)*	249 (27.6)
*>20,000 (USD 266.91)*	108 (12.0)
*Don’t Know*	21 (2.3)
Vegetarian (self-reported)	152 (16.9)
Height (cm), mean (SD)	153.9 (5.6)
Weight (kg), mean (SD)	48.9 (8.8)
BMI (kg/m^2^), mean (SD)	20.6 (3.6)
Mid-arm circumference (mm), mean (SD)	24.3 (3.1)
Haemoglobin, mean (SD)	11.1 (1.3)
Gestation age at delivery (Weeks), median (IQR) *	39.0 (38.0, 40.0)
Preterm (Gestation age < 37 weeks)	61 (6.8)
Children	*n* = 902 Live singleton births
Male sex	477 (52.9)
Birth weight (grams), median (IQR)	2740.0 (2500.0, 3011.0) (*n* = 880)
Birth length (cm), median (IQR)	47.2 (46.1, 48.5) (*n* = 823)
Head circumference (cm), median (IQR)	34.0 (33.0, 34.5) (*n* = 823)
Apgar score at 1 min, median (IQR)	7.0 (6.0, 7.0) (*n* = 748)
Apgar score at 5 min, median (IQR)	8.0 (8.0, 8.0) (*n* = 751)

* *n* = 880.

**Table 2:** Feeding Practices.

Feeding Practices	*n* = 902 *n* (%)
Early initiation of Breastfeeding (within 1 h) Not available	703 (77.9) 32 (3.5)
Reason for delay of Early initiation of breastfeeding (*n* = 167) Breastmilk was not expressed within 1 h of birth Mother was shifted to the ward after 1 h of delivery After delivery, baby was shifted to NICU Others * Reason not available	38 (4.2) 63 (6.9) 29 (3.2) 31 (3.5) 6 (0.8)
Colostrum fed to the baby	859 (95.2)
Baby given pre-lacteal feed	77 (8.5)
Kind of pre-lacteal feed (*n* = 77) Cow’s Milk Dates Ghutti Gripe Water Honey Sugar Water Warm Water Water Not available	1 (1.3) 1 (1.3) 1 (1.3) 1 (1.3) 35 (45.5) 31 (40.3) 1 (1.3) 1 (1.3) 5 (6.5)
Mode of feeding prior to hospital discharge Exclusive Breast Milk Mixed Feeding Exclusive formula feeding Expressed milk Any other Not available	843 (93.5) 9 (1.0) 11 (1.2) 1 (0.1) 1 (0.1) 37 (4.1)

* Baby was handed to the mother after 1 h of delivery; baby was not able to suck; mother was unconscious; mother was having medical complication; religious ritual.

**Table 3:** Association of exclusive breastfeeding at 6 months with mother and child characteristics.

Mother and Child Characteristics		Exclusive Breastfed at 6 Month					
		Yes	No	*p*-Value	Relative Risk (95% CI)		
		427	417		Unadjusted	Adjusted *	Adjusted **
Mother’s characteristics							
Mother’s age							
*18–20*	197	97 (49.2)	90 (45.7)	0.51	1.0	1.0	1.0
*21–25*	461	218 (47.3)	209 (45.3)		0.98 (0.83, 1.16)	1.00 (0.84, 1.21)	1.00 (0.83, 1.21)
*26–30*	208	97 (46.6)	98 (47.1)		0.96 (0.79, 1.17)	1.00 (0.81, 1.25)	1.01 (0.81, 1.26)
*31–35*	38	15 (41.7)	20 (55.6)		0.83 (0.55, 1.24)	0.90 (0.56, 1.3)	0.84 (0.55, 1.28)
Mother’s education							
*≤Secondary*	467	241 (51.6)	196 (42)	0.006	1.21 (1.05, 1.38)	1.15 (1.00, 1.32)	1.16 (1.01, 1.33)
*Senior Secondary or Professional*	435	186 (42.8)	221 (50.8)		1.0	1.0	1.0
Monthly Income (INR)							
*<10,000*	524	251 (47.9)	238 (45.4)	0.61	1.0	1.0	1.0
*10,000–20,000*	249	112 (45)	123 (49.4)		0.93 (0.79, 1.09)	0.95 (0.81, 1.11)	0.95 (0.82, 1.11)
*>20,000*	108	52 (48.1)	48 (44.4)		1.01 (0.82, 1.25)	1.20 (0.97, 1.48)	1.22 (0.99, 1.51)
BMI (kg/m^2^), mean (SD)		20.3 (3.2) (*n* = 427)	21.1 (3.9) (*n* = 417)	0.002	0.97 (0.95, 0.99)	0.97 (0.95, 0.99)	0.97 (0.95, 0.99)
Parity (no. of live children)							
*Multiparous*	469	239 (51)	202 (43.1)	0.029	1.16 (1.01, 1.33)	1.21 (1.05, 1.41)	1.23 (1.06, 1.43)
*Nulliparous*	*433*	188 (43.4)	215 (49.7)		1.0	1.0	1.0
Type of Delivery							
*Vaginal normal/instrument*	551	288 (52.3)	242 (43.9)	0.005	1.23 (1.06, 1.42)	1.17 (1.01, 1.37)	1.18 (1.01, 1.37)
*Caesarean*	329	139 (42.2)	175 (53.2)		1.0	1.0	1.0
Child characteristics							
Hospitalisation at 1, 6 or 12 months							
*Yes*	21	6 (28.6)	13 (61.9)	0.044	0.62 (0.32, 1.2)	0.60 (0.31, 1.15)	0.59 (0.30, 1.16)
*No*	874	421 (48.2)	404 (46.2)		1.0	1.0	1.0

* adjusted only for the treatment group. ** values reported adjusted for each factor and treatment group.

**Table 4:** Association of exclusively breastfeeding at 6 months with child weight, height and head circumference at 6 and 12 months.

	Exclusive Breastfed at 6 Months		*p*-Value	Difference (95% CI)	β Coefficient * (95% CI)
	Yes	No			
Weight (grams) at 6 months, mean (SD)	6956.5 (854.7)	6882.2 (911.3)	0.22	74.2 (−45.1, 193.6)	91.8 (−29.9, 213.5)
Height (cm) at 6 months, mean (SD)	64.6 (2.8)	64.7 (2.9)	0.74	−0.07 (−0.46, 0.32)	0.004 (−0.39, 0.4)
Head Circumference (cm) at 6 months, mean (SD)	42.0 (1.4)	42.1 (1.4)	0.19	−0.13 (−0.31, 0.06)	−0.07 (−0.26, 0.12)
Weight (grams) at 12 months, mean (SD)	8415.1 (1022.4)	8392.7 (1024.1)	0.75	22.4 (−116.3, 161.1)	56.5 (−83.2, 196.2)
Height (cm) at 12 months, mean (SD)	72.2 (3.0)	72.4 (3.3)	0.44	−0.17 (−0.59, 0.26)	−0.074 (−0.51, 0.36)
Head Circumference(cm) at 12 months, mean (SD)	44.6 (1.3)	44.6 (1.4)	0.30	−0.10 (−0.28, 0.09)	−0.04 (−0.23, 0.14)

Difference calculated as those who exclusively breastfed at 6 months minus those who did not breastfeed. * Adjusted for the treatment group, mother age, mother education, household income, mother BMI, parity and delivery type.

**Table 5:** Association of the Diet Diversity (DD) score with the mother’s characteristics.

		DD	*p*-Value
	*n*	mean (SD)	
Overall	878	4.66 (1.15)	
Mother’s age, years			0.335
*18–20*	193	4.53 (1.13)	
*21–25*	448	4.68 (1.18)	
*26–30*	202	4.73 (1.10)	
*31–35*	35	4.71 (1.15)	
Mother’s education			
*≤Secondary*	427	4.62 (1.14)	0.218
*Senior Secondary or Professional*	451	4.71 (1.16)	
Monthly Income (INR)			
*<10,000*	507	4.55 (1.16)	0.002
*10,000–20,000*	244	4.76 (1.09)	
*>20,000*	106	4.93 (1.17)	
Parity (no. of live children)			
*Multiparous*	455	4.65 (1.13)	0.655
*Nulliparous*	423	4.68 (1.17)	
Type of Delivery			
*Vaginal normal/instrument*	537	4.68 (1.15)	0.414
*Caesarean*	319	4.61 (1.15)	
Exclusive breastfed			
*Yes*	425	4.65 (1.15)	0.803
*No*	414	4.67 (1.16)	

## Data Availability

The data will be made available upon receiving written request and approval granted by the study’s Mentoring and Advisory Committee chaired jointly by Prof Reynaldo Martorell (USA) and Srinath Reddy (India).

## Supplementary Materials

The following supporting information can be downloaded at: https://www.mdpi.com/article/10.3390/ijerph19095088/s1, Box S1: Food groups covered in the Food Frequency Questionnaire (FFQ); Box S2: Definitions of IYCF Indicators. Figure S1. CONSORT Diagram.
