# Supplementary material for: Infant Young Child Feeding Practices in an Indian Maternal–Child Birth Cohort in Belagavi, Karnataka
Source: Int J Environ Res Public Health. 2022 Apr 22;19(9):5088. doi: 10.3390/ijerph19095088 (PMC9104747; doi:10.3390/ijerph19095088)
Supplement: Supplementary file 1 [file ijerph-19-05088-s001.zip › ijerph-1636244-supplementary.pdf]

Supplementary Materials

Box S1. Food groups covered in the Food Frequency Questionnaire (FFQ).

- Meat [lamb, mutton, goat, veal, rabbit, beef, pork; their curries]
- Poultry [chicken, turkey, duck, their curries]
- Organ meats [liver, kidney, brain, spleen, heart, sausages, nihari, paya]
- Fish [fresh-water and sea-water fish; preserved fish such as salted fish, canned fish, dried fish]
- Shellfish and crustaceans [crab, squid, prawns, molluscs, caviar]
- Eggs [Includes preserved eggs, duck eggs]
- Milk and milk products: [milk, cottage cheese, processed cheese, curd, raita, lassi, buttermilk and other milk-based drinks]
- Milk-based desserts [custard, khoya, firni, kheer, milk puddings, rasgullah/rasmalai, ice cream, shrikhand, mishit dahi] all milk based desserts
- Deep fried foods: western style [french fries, potato chips, onion rings, chicken nuggets]
- Deep fried foods: Indian style [samosas, papad, pakoras, sev, namak paray, egg rolls, poori, kachori]
- Western style desserts/sweet snacks [cakes; pies; chocolate; candy; biscuits]
- Mithai [burfi/ladoo; gulab jamun; halwa, panjiri/gondh ladoo]
- Cold beverages [carbonated beverages, sherbets, energy drinks and other soft drinks]
- Fruits (1) [strawberries, pineapples, jumbo berries (jamuns), apples]
- Fruits (2) all seasonal fruits except the ones above
- Fruit juices [any type, homemade, purchased, fresh, frozen]
- Nuts/seeds [Includes peanuts, sunflower seeds]: Walnuts, almonds, cashew nuts are given separately
- Leafy greens [all fresh leafy green vegetables: spinach, mustard or turnip greens; asparagus either raw or cooked]
- Other raw vegetables [any raw vegetables not included in the preceding categories]
- Legumes and pulses [includes all daals, chickpeas, lentils, sprouted whole pulses, soybean nuggets, soya chap]
- Use of pickles, pickled foods [achar, chutneys, pickled vegetables, jams, jellies, marmalades, etc.]
- Other cooked vegetables [any cooked vegetables not included in the preceding categories]
- Refined cereals with less fibre [boiled rice, fried rice, biryani, pulao, idli, dosa, semolina,sago, pearl barley, pasta, sheermal, taftan, white bread slice]
- Whole grain (cereal dished with more fibre) [Roti made with whole meal flour, brown rusk, whole wheat porridge, bread slice whole meal/brown] and Millets like jowar, bajra, ragi, etc.
- Tea consumption [black tea, tea with milk and sugar and any other tea like green tea]
- Coffee consumption [coffee with and without milk and/sugar.
- Butter spread (all types of butter used)
- Other things consumed (other)

Box S2. Definitions of IYCF Indicators.

Indicators	Definition	Rationale for Indicator
Early initiation of breastfeeding (EIBF)*	Percentage of children born in the last 24 months who were breastfed within one hour of birth.	Provides short-term and long-term benefits, promotes skin-to-skin contact between mother and baby.
Exclusive breastfeeding under six months (EBF)*	Percentage of infants of 0-5 months age who were exclusively breastfed in the previous day.	Safest and healthiest option, reduces mortality and morbidity, protects against infectious and non-communicable diseases.
Continued breastfeeding (CBF)*	Percentage of children of 12-23 months age who received breastmilk in the previous day.	Helps to meet energy requirements, protects against various illnesses, promotes brain development.
Introduction of solid, semi-solid or soft foods (ISSSF)/ Complementary feeding (CF)*	Percentage of infants of 6-8 months age who received solid, semi-solid or soft food in the previous day.	Helps to meet increased nutritional requirements of infants that cannot be met by breastfeeding alone.
Minimum dietary diversity (MDD)*	Percentage of children of 6-23 months age who consumed at least five out of eight defined food groups in the previous day.	Protects against micronutrient deficiencies.
Minimum meal frequency (MMF)*	Percentage of children of 6-23 months age who consumed solid, semi-solid or soft foods (but also including milk feeds for non-breastfed children) the minimum number of times in the previous day. The minimum number refers to:<br>• 2 feedings of solid, semi-solid or soft foods for breastfed infants aged 6-8 months<br>• 3 feedings of solid, semi-solid or soft foods for breastfed children aged 9-23 months<br>• 4 feedings of solid, semi-solid or soft foods or milk feeds for non-breastfed children aged 6-23 months where at least one feed must be a solid, semi-solid or soft feed.	Helps to meet energy and other nutritional requirements. Protects against growth faltering, stunting, wasting, micronutrient deficiencies, etc.

Minimum milk feeding frequency for non-breastfed children (MMFF)*	Percentage of non-breastfed children of 6-23 months age who received at least two milk feeds during the previous day.	Helps to meet nutritional requirements.
Minimum acceptable diet (MAD)*	Percentage of children of 6-23 months age who consumed a minimum acceptable diet in the previous day. Minimum acceptable diet refers to:<br>• For breastfed children: those who received MDD and MMF on the previous day<br>• For non-breastfed children: those who received MDD, MMF and MMFF on the previous day.	Helps to meet energy and other nutritional requirements.
Egg and/or flesh food consumption (EFF)*	Percentage of children of 6-23 months of age who consumed egg and/or flesh food during the previous day.	Helps to meet nutritional requirements especially energy, protein, essential amino acids, important fatty acids, vitamin B12, A, D, E, phosphorus, calcium, zinc, etc. Promotes linear growth.
Sweet beverage consumption (SwB)#	Percentage of children of 6-23 months of age who consumed a sweet beverage during the previous day.	Usually provides empty calories and may replace more nutritious foods in the diet.
Unhealthy food consumption (UFC)#	Percentage of children of 6-23 months of age who consumed selected sentinel unhealthy foods during the previous day.	These foods are high in sugar, salt, unhealthy fats and refined carbohydrates, energy-dense and nutritionally poor.
Zero vegetable or fruit consumption (ZVF)#	Percentage of children of 6-23 months of age who did not consume any vegetable or fruit during the previous day.	Associated with increased risk of non-communicable diseases.
Bottle feeding (BoF)#	Percentage of children of 6-23 months of age who were from a bottle with a nipple during the previous day.	Must be avoided since bottles are difficult to clean, act as a rote of pathogen transmission and may interfere with optimal suckling behaviour.

*Healthy practices, #Unhealthy practices.

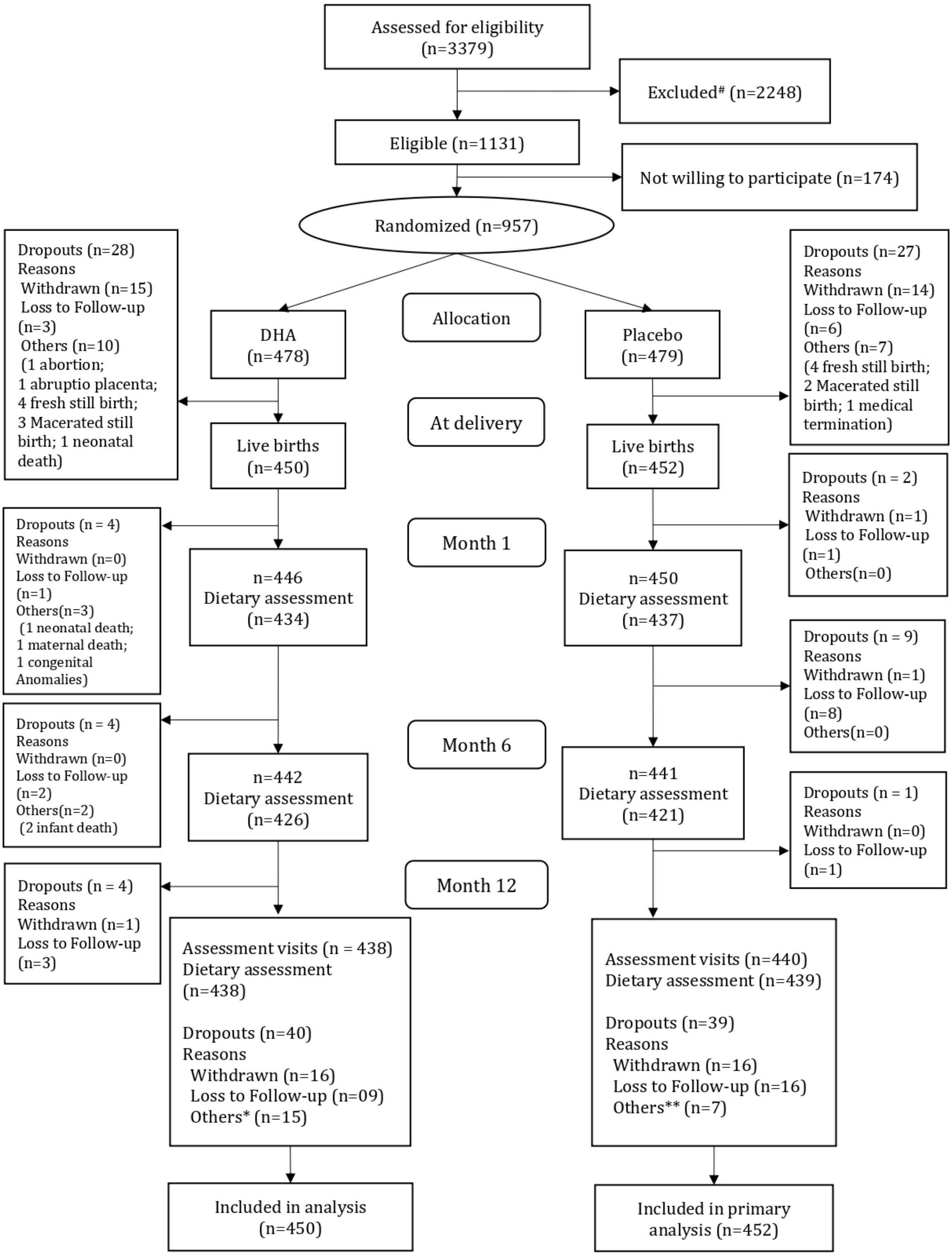

Supplementary Figure S1. Consort. # Reasons for exclusion: gestational diabetes (n = 69); Hb < 7 gm% (n = 46); gestational age >20 weeks (n = 673); high-risk pregnancies (n = 118); chronic conditions (n = 246); under any other trial (n = 4); delivery plan other than PK (n = 835); missing/wrong contact information (n = 257). * Others included: abortion (n = 1); abruptio placenta (n = 1); fresh stillbirth (n = 4); macerated stillbirth (n = 3); neonatal death (n = 2); maternal death (n = 1); congenital anomalies (n = 1); infant death (n = 2) in DHA group. ** Others included: fresh stillbirth (n = 4); macerated stillbirth (n = 2); medical termination (n = 1) in Placebo group.
